# Food Safety Knowledge, Attitudes, and Behaviors of Native American Families with Young Children: A Mixed Methods Study

**DOI:** 10.1007/s40615-015-0190-z

**Published:** 2015-12-22

**Authors:** Kara Vlasin-Marty, Paula Ritter-Gooder, Julie A. Albrecht

**Affiliations:** Department of Nutrition and Health Sciences, University of Nebraska-Lincoln, 119E Leverton Hall, Lincoln, NE 68583 USA

**Keywords:** Food safety, Health belief model, Foodborne illness, Food handling practices, Mixed methods, Native American

## Abstract

Children are at increased risk for foodborne illness due to underdeveloped immune system. Limited research has been reported on food safety knowledge of Native American families with children 10 years of age and younger. This study was conducted to determine the food safety knowledge, attitudes, and behaviors of the main food preparer in these families by collecting quantitative and qualitative data simultaneously in a mixed method approach. A food safety knowledge survey created using FightBAC!^™^ concepts was administered prior to focus groups discussions held in Native American communities using a script based upon the Health Belief Model. Quantitative data were analyzed using SPSS. Qualitative data were coded by three reviewers independently and then compared jointly for themes. Over three fourths of participants (*n* = 102) were female with an average age of 38.3 years. Over one half of participants were unemployed (54 %), lived on reservations (54 %), and 86 % had a high school degree or higher level of education. The following four themes emerged from the eight focus groups (*n* = 66): food can make one sick, I am not in control when others handle food, I know how to safely prepare foods for my family, and I do not have time or best equipment (for food safety). Mixed method analysis revealed that participants were aware of the severity and susceptibility for foodborne illness but were confident in preparing foods safely for their family. A food safety education program for Native American food preparers with young children is needed to prevent foodborne illness (FBI) in this population and promote safe food handling practice.

## Introduction

The incidence of foodborne illness (FBI) cases, hospitalizations, and deaths continues to be an issue in the USA. In 2011, FBI estimates using FoodNet, the surveillance system that tracks trends of the most common infections caused by FBI within the USA, were published [1]. About one in six (or 48 million) people get sick each year from contaminated food, with 128,000 hospitalizations and 3000 deaths occurring annually. In addition, a higher incidence of *Campylobacter*, *Cryptosporidium*, *Escherichia coli* O157, *E. coli* non-O157, *Salmonella*, *Shigella*, and *Yersinia* infection rates were found in children under 5 years of age than any other age group. These FBIs can lead to long-term health problems, death of infants and children, and an estimated cost of 2.3 billion dollars a year. Many cases of FBI can be avoided if proper food safety procedures are followed. It is necessary to focus food safety education on the main food preparer for young children to decrease the incidence, morbidity, and mortality of FBI and ultimately decrease associated costs of treating the illness. For consumers, the Partnership for Food Safety Education has developed the following four core messages: clean, separate, cook, and chill [2]. Clean focuses on hand hygiene, food contact surfaces, and washing produce. Separate refers to cross contamination from improperly washed hands or infected hands and food contact surfaces. Inadequate cooking foods, use of thermometers to determine doneness, and properly cooling and reheating foods are practices within the cook concept. Practices for the chill concept include proper cooling foods, proper thawing foods, cold food storage, and handling leftovers.

Numerous studies have been conducted to determine food safety knowledge, attitudes, and behaviors of different populations. Studies among families [3, 4] and mothers with children [5, 6] have been reported. Main food preparers in families with young children in the general population of the USA [7, 8] and in the Latino population have also been studied [9]. In a study among food safety professionals, the unfamiliarity with some culturally specific foods and lack of culturally specific food safety resources were identified as missing gaps for educating ethnic populations and surveying their food environments [10]. In a report of child poverty, they indicated that people living in high-poverty areas experience higher rates of foodborne illness and children in these low-income communities may be at greater risk for foodborne disease particularly salmonellosis and shigellosis [11]. In this report, ethnicity (black and Native American) did play a role in in higher injury risk for fire, suffocation, poisoning, falls, motor vehicles, and firearms for children. Besides low income as a factor for increased risk of foodborne illness, limited data on specific ethic groups have been reported [11].

The Health Belief Model (HBM) [12, 13] explains why people reject screening tests and preventive health-care measures for diseases without symptoms and provides a framework to design strategies for behavior change. The HBM assesses an individual’s perceived threat posed by a health issue, benefits of avoiding that threat, and factors which influence their decision to act [13, 14].

No research has focused solely on the food safety knowledge, attitudes, and practices of Native Americans. Researchers who have studied Native American communities recommend that research should be conscious and respectful of Native American culture, beneficial to the participants, and involve a mutual relationship of trust between communities and researchers [15]. A mixed method study using qualitative inquiry which honors the worldview of many Native Americans and quantitative data collection is ideal for understanding food safety among this population [16]. The purpose of this mixed method study was to determine the food safety knowledge and explore attitudes and behaviors of the main food preparer in Native American families with young children ≤10 years of age. Food safety knowledge was compared to attitudes and behaviors to inform future food safety education within this population.

## Methods

A convergent parallel mixed method design used in previously conducted research studies among families with young children was employed [3, 8]. Both qualitative and quantitative data were collected in parallel from the same Native American families, analyzed separately, and then merged together.

### Quantitative

A food safety knowledge survey was developed using food safety concepts (clean, separate, cook, and chill) from the FightBac!^™^ and Be Food Safe^™^ [17] campaigns and a previously validated survey [18]. The modified survey was validated with the population [19]. The survey consisted of true, false, and multiple choice questions based on recommended food safety practices and demographic questions. Convenience sampling and the snowball technique [20] were used to recruit individuals for pilot testing the survey. To establish trust and consideration for traditional beliefs [21], researchers and extension educators identified key community individuals who recruited Native Americans (*n* = 38, pilot test) living on or off reservations who were main food preparers for children ≤10 years of age. After informed consent, the survey instrument was administered in community centers. A $5 dollar retail gift card was given upon completion of the survey. Based upon participant feedback from the pilot survey and content review by food safety experts and professionals, the number of knowledge questions was reduced from 41 to 29 to lower respondent burden for the final survey. Data from the pilot survey (*n* = 38) for the 29 questions were combined with the data from the mixed method study (*n* = 52) for analyses. Approval for all phases of the study was obtained from the Institutional Review Board prior to beginning the study (IRB nos. 20120112245EP and 20120412564EX)

### Qualitative

A focus group script used in other food safety studies among families with young children [3, 8] was followed. It consisted of questions to elicit information about the meaning of food safety and FBI among participants. The script was based upon the constructs of the HBM [13] for exploring the participants’ perceptions of severity, susceptibility, benefits, barriers, cues to action, and self-efficacy related to FBI. Other questions addressed current food handling practices, cultural beliefs related to food safety, food safety information sources, and preferred methods of receiving food safety information. Initial questions elicited information about frequently prepared foods to gain understanding of the common foods and ingredients used in this population and assist participants with feeling comfortable and talking within the group.

### Subject Recruitment

Recruitment for the mixed method research followed the same process described for the pilot test as stated in the “Quantitative” section. In addition, recruitment posters were placed at sites and community centers where focus group discussions were to be conducted. Of eight focus groups conducted (including one pilot focus group), five were held on reservations.

### Mixed Method Research

Research was conducted in local community centers both on and off reservations. After verbal confirmation of being a main food preparer in a Native American home with children 10 years of age or younger, signed informed consent was provided and the food knowledge survey was administered in the group setting. After the surveys were completed, one researcher conducted all the group discussions using the focus group script [22, 23]. Discussions were audio taped and lasted from 45 to 90 min. Notes were recorded by research team observers who sat with the interviewer. At the completion of the study, participants received a $25 retail gift card. Focus groups were conducted until saturation, and common themes emerged (*n* = 8).

### Quantitative Analysis

Food safety knowledge responses, including those from the pilot study, were analyzed using SPSS (SPSS version 21, SPSS Inc., Chicago, IL, 2012). All correct responses were coded as one point each, including those questions containing multiple correct responses. Levene’s test for equality of variances was used to determine if there was a significance difference between those living on or off the reservation. Individual responses, subcategories of the survey (FightBac!^™^ concepts clean, cook, chill, and separate, foods and groups at increased risk), and the survey as a whole were scored. Cronbach’s alpha was measured to determine internal consistency.

### Qualitative Analysis

The focus group discussions were transcribed, and observation notes were added. Transcriptions were analyzed independently by three researchers trained in qualitative data analysis [20]. The HBM constructs were used as a codebook or guide for analysis. First, the transcribed data were reviewed in entirety and memos or ideas were written alongside the data. Then, segments of text were divided and codes were assigned to describe content. Next, the codes were grouped into themes. Finally, the researchers came together to pool the themes and used a minimum of two-third interrater agreement to reach final analysis.

### Mixed Method Analysis

Themes derived from the focus groups were compared to the food knowledge survey responses to uncover similarities and differences between the two data sets [16].

## Results

### Participant Demographics

Over three fourths of participants (*n* = 102) were female (Table 1) with an average age of 38.3 years. Over one half of participants were unemployed (54 %) and lived on reservations (54 %). The majority of participants had education beyond high school (86 %).

**Table 1:** Demographic characteristics of main food preparers in Native American families with children ≤10 years of age participating in mixed method study on food safety and foodborne illness

	Pilot study *n* = 36	Focus group *n* = 66	Total *n* = 102
Gender, *n* (%)			
Male	4 (11.1)	15 (22.7)	19 (18.6)
Female	32 (88.9)	51 (77.3)	83 (81.4)
Age, years			
(Mean ± SD)	38.4 ± 14	38.3 ± 14	38.3 ± 13.9
Number of children			
(Mean ± SD)	2.5 ± 1.1	2.4 ± 1.3	2.4 ± 1.3
Age of children, years			
(Mean ± SD)	5.2 ± 3.0	5.1 ± 2.7	5.2 ± 2.8
Employment, *n* (%)			
Full-time	22 (61.1)	13 (19.7)	35 (34.3)
Part-time	4 (11.1)	9 (13.6)	13 (12.7)
Unemployed	10 (27.8)	44 (66.7)	54 (53.0)
Residency, *n* (%)			
Reservation	16 (44.4)	38 (57.6)	54 (53.0)
Non-reservation	20 (55.6)	28 (42.4)	48 (47.0)
Education, *n* (%)			
Less than high school	2 (5.5)	0 (0)	2 (2.0)
Some high school	0 (0)	12 (18.2)	12 (11.8)
High school/GED	9 (25.0)	23 (34.8)	32 (31.4)
Additional training beyond high school	5 (13.9)	4 (6.1)	9 (8.8)
Some college	13 (36.1)	16 (24.2)	29 (28.4)
College graduate	6 (16.7)	10 (15.2)	16 (15.6)
Post-college graduate	1 (2.8)	1 (1.5)	2 (2.0)
Tribal affiliation^a^, *n* (%)			
Omaha	13 (36.1)	33 (55.0)	46 (45.1)
Santee Sioux	13 (36.1)	8 (12.10	21 (20.6)
Winnebago	1 (2.8)	13 (19.7)	14 (13.7)
Ponca	1 (2.8)	2 (3.0)	3 (2.9)
Yankton Sioux	1 (2.8)	2 (3.0)	3 (2.9)
Rosebud Sioux	1 (2.8)	1 (1.5)	2 (2.0)
Dakota	1 (2.8)	0 (0.0)	1 (1.0)
Oglala Sioux	0 (0.0)	2 (3.0)	2 (2.0)
Cheyenne	1 (2.8)	1 (1.5)	2 (2.0)
Cheyenne River Sioux	0 (0.0)	1 (1.5)	1 (1.0)
Northern Cheyenne	0 (0.0)	1 (1.5)	1 (1.0)
Gros Ventre	0 (0.0)	1 (1.5)	1 (1.0)
Sisseton Wahpeton	0 (0.0)	1 (1.5)	1 (1.0)
Lakota	0 (0.0)	1 (1.5)	1 (1.0)
Sioux	0 (0.0)	1 (1.5)	1 (1.0)
Crow Creek	1 (2.8)	0 (0.0)	1 (1.0)
Arapaho	1 (2.8)	0 (0.0)	1 (1.0)
Missing response	4 (11.1)	8 (12.1)	12 (11.8)

^a^Participants may have indicated more than one tribal affiliation. Missing responses for pilot = 4, focus group = 8

### Quantitative Results

Results of the knowledge survey are provided in Table 2. The average score on the knowledge survey was 62.2 %. The food safety knowledge score between the reservation and non-reservation residents was not significant (*p* = 0.388). A significant difference between those living on or off the reservation was found in two questions, “which foods need to be refrigerated to prevent food poisoning” (*p* = 0.042) and “how should fresh fruits and vegetables be washed to keep you from getting food poisoning” (*p* = 0.044). Reliability testing produced a Cronbach’s alpha of 0.650.

**Table 2:** Rank order, by category and within question, of correct food safety knowledge responses of Nebraska Native American primary food preparers (*n* = 102) for children ≤10 years of age

Question	Correct response	*n* (%)^a^
Clean		
How should you wash fresh fruits and vegetables to keep you from getting food poisoning?	Hold under cool running water	87 (85.3)
Washing hands after changing a diaper	Decreases the chance of food poisoning	79 (77.5)
How should dishes be washed to prevent food poisoning? (check all that apply)	Hand wash them and rinse right after the meal and then let them air dry	65 (63.7)
	Wash and dry them in a dishwasher	55 (53.9)
Which is an acceptable way to clean a cutting board or counter after it is used for raw meat? (check all that apply)	Wash with hot soapy water, rinse with water, and then rinse with bleach	63 (61.8)
	Washing cutting board in a dishwasher	34 (33.3)
What is the best way to wash your hands?	Run water, moisten hands, apply soap, rub hands together for 20 s, rinse hands, and dry hands	52 (51)
How should kitchen counters be cleaned to prevent food poisoning?	Wash with hot soapy water, rinse, and wipe with a bleach solution	24 (23.5)
Separate		
When preparing food, you should wash your hands after touching which of these? (check all that apply)	Dirty pots and pans	92 (90.2)
	Cell phone or home telephone	88 (86.3)
	Fresh fruit	42 (41.2)
If you have a cut or sore on your hand, what should you do before you prepare food for your family?	Wash hands, put a bandage on the sore, and wear a glove	86 (84.3)
Putting raw meat in a separate bag (away from other food items) before placing it in the grocery cart	Decreases the chance of food poisoning	82 (80.4)
Where is the best place to store raw meat in the refrigerator?	Below ready-to-eat foods, like salad	62 (60.8)
Cook		
A food is properly cooked in a microwave oven when (check all that apply)	You follow directions on the package	82 (80.4)
	You test the food with a thermometer	49 (48.0)
What is the best way to tell when chicken has cooked long enough?	Test with a meat thermometer	38 (37.3)
What is the best way to tell if hamburgers are cooked enough to prevent food poisoning?	Measure the temperature with a food thermometer	37 (36.3)
To prevent food poisoning, how long should leftover soup be heated?	Until it is boiling hot	36 (35.3)
Chill		
It is safe to give an infant a bottle of baby formula that has been out of the refrigerator for longer than 2 h?	False	90 (88.2)
Which food needs to be refrigerated to prevent food poisoning?	An open can of corn	80 (78.4)
If a leftover food looks and smells good, it is still safe to eat	False	78 (76.5)
Your child is going to be eating 2 h after you cook a meal. How should you keep the meal safe before your child eats it?	Store it in the refrigerator and reheat it when the child is ready to eat it	64 (62.7)
How long can you store raw hamburger and chicken in the refrigerator to eat later?	1–2 days	68 (66.7)
Refrigeration eliminates harmful germs in food.	False	59 (57.8)
Your electricity went off in your freezer, and the meat, chicken, and fish thawed and felt warm. What should you do to prevent food poisoning?	Throw them away	51 (50.0)
What is the safest way to cool a large pot of hot soup?	Put the soup in a clean shallow pan and refrigerate right away	17 (16.7)
How long can you store cooked hamburger and chicken in the refrigerator to eat later?	3–4 days	16 (15.7)
Foods that increase risk		
Undercooked chicken and raw eggs can carry *Salmonella* (a harmful germ)	True	101 (99.0)
*E. coli* (a harmful germ) in undercooked hamburger can cause kidney failure in children	True	88 (86.3)
It is safe to use raw eggs in recipes that will not be cooked	False	83 (81.4)
Eating which of these foods will increase a person’s risk of food poisoning? (check all that apply)	Hamburger cooked rare	88 (86.3)
	Sushi	73 (71.6)
	Raw milk (not pasteurized) or fresh cheese made with raw milk	66 (64.7)
	Raw homemade cookie dough or cake batter	63 (61.8)
	Baked potato that was left on the counter overnight	61 (59.8)
	Fried eggs with a runny or soft yolk	60 (58.8)
	Raw shellfish	50 (49.0)
	Milk with raw egg added	50 (49.0)
	Unpasteurized fruit juice	43 (42.2)
	Infant milk or formula with honey added	38 (37.3)
	Leftover soup reheated until warm but not boiling	35 (34.3)
	Raw sprouts (alfalfa, bean, clover, and radish)	31 (30.4)
	Soup cooled on the counter	29 (28.4)
	Sliced melons or cantaloupe	15 (14.7)
Groups at increased risk		
Which of these people will likely get sick from harmful germs in food? (check all that apply)	Older people (age 60 and over)	94 (92.2)
	Pregnant women	93 (91.2)
	Preschool children	91 (89.2)
	People with type 2 diabetes	69 (67.6)
	Cancer patients	68 (66.7)
Which foods will likely cause food poisoning for pregnant women, infants, and children? (check all that apply)	Raw eggs	91 (89.2)
	Undercooked eggs	78 (76.5)
	Hot dogs that have not been heated	65 (63.7)
	Cold potato salads	19 (18.6)

^a^Survey composite score = 62.2 %

For the clean concept, most participants knew how to wash fresh fruits and vegetables (85 %) and to correctly wash hands after changing a diaper (77 %), but only 51 % of the participants correctly knew how to correctly wash their hands and fewer (24 %) did not know how to properly clean their counters. All the questions in the separate concept were correctly answered by more than 50 % of the participants except only 41 % knew to wash their hands after handling fresh fruit. In the cook concept, most of the participants (80 %) follow the package directions for microwaveable foods. Only 37 and 36 %, respectively, use a thermometer to determine the doneness of chicken or hamburger. Most of the questions in the chill concept were answered correctly by more than 50 % except for questions on proper cooling (17 %) and how long to store cooked meat and poultry ((16 %) (Table 2).

Foods that participants did not recognize as increasing the risk of FBI (less than 50 % identified it) were eggs with a runny yolk (49 %), milk with raw egg (49 %), unpasteurized fruit juice (42 %), infant formula with added honey (37 %), leftover soup not heated to boiling (34 %), raw sprouts (30 %), soup cooled on the counter (28 %), and sliced melons (15 %) (Table 2).

### Qualitative Results

Themes with supporting quote codes are provided in Table 3. The following four themes emerged: food can make one sick, I am not in control when others handle food, I know how to safely prepare foods for my family, and I do not have time or best equipment (for food safety).

**Table 3:** Qualitative analysis of focus group (*n* = 8) discussions on foodborne illness and food safety among primary food handlers in Native American families with children ≤10 years of age

Theme	Quotes
Food can make one sick	“I ate them and I got sick right away and a couple hours later you know I just got really sick… I think it was from that food because as soon as I ate it my stomach started hurting.”
	“As soon as I got done eating, I started throwing up.”
	“If someone probably got sick from chicken in the house, stop cooking chicken for a while.”
	“Well with my mom, sugar makes her more sick, because she is diabetic.”
	“… like Native Americans, a lot of them are lactose intolerant.”
	“Keep a close eye on the child maybe; make sure there was not a reaction.”
	“Some kids are allergic to it (honey).”
	“I know someone who passed away… *E. coli*… she got ice cubes out of a cooler that they were keeping hamburger patties in and it shut her kidneys down. She was not much older, about eight.”
I am not in control when others handle food	“That is why we do not go out to eat… you do not know what they are going to put in your food. Cause there is a lot of people handling the food. It is not as processed and stuff.”
	“I would rather do it myself and make sure it is done right.”
	“… they may not cook it right.”
	“… we have so many fast food chains and restaurant and who knows if they do what they are supposed to do properly…”
	“like chicken soup they have at the feast, I would not eat it in the summer. I got sick off of it once so I would not eat chicken soup.”
	“cause you do not know how long it (food) sits cause at feasts they will be make it in the morning and then it will sit all day.”
I know how to safely prepare foods for my family	“You know they always say you need a meat thermometer, but I mean just by looking at it, you can tell if it is red or pink.”
	“Cause at home, you are the one preparing the food, making sure it is clean, you pay attention to how clean your stuff is…”
	“I watch how I pack my meat and clean my kitchen… I am pretty confident.”
	“The things I can make I am confident that it is ok.”
	“Cause nobody has ever gotten sick from my cooking.”
	“Cause you know like none of my kids ever got sick from it.”
	“I have a cleaning solution, half water, half bleach.”
	“I just put in a couple of drops (bleach).”
I do not have time or best equipment	“Some days you are in a rush you know trying to get food out there quickly, maybe take a shortcut some days, rushing to get water boiling over here and do not have time to wash your hands again, kids are crying, phones ringing, there is always something.”
	“You just do not feel like cleaning up everything right away, you are in a hurry…” (food may be left out too long)
	“I would say time… if I am in a rush I forget to wash sometimes…”
	“When I watch the cooking channels the stuff for everything they use to cook, at home I only have the basic stuff.” (refrigeration space is needed for proper storage of large quantities of food)
	“A nicer fridge would help. It would work better.”
	“… at fast food restaurants, there is a lot of stuff I do not have… stuff for countertops… I do not have that.”

### Mixed Method Results

**Fig. 1:**
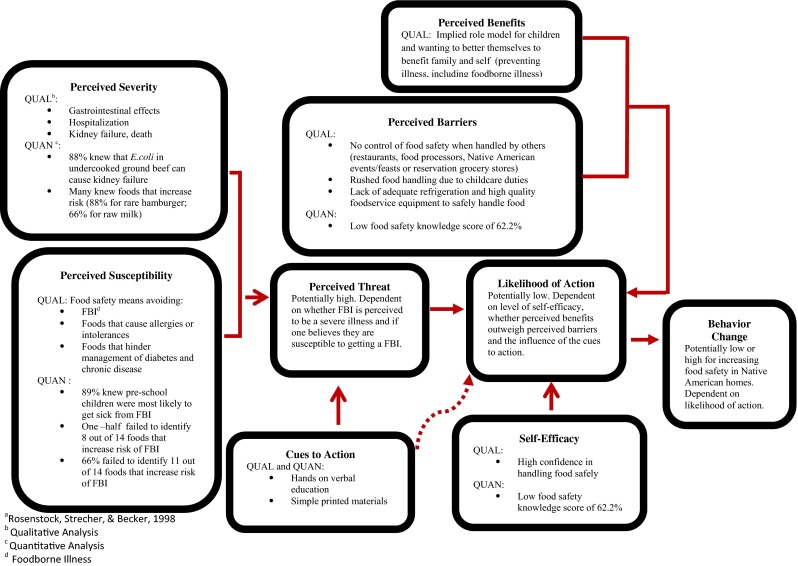
Mixed method analysis of food safety knowledge, attitudes, and beliefs of main food preparers in Native American families with children ≤10 years of age through the lens of the Health Belief Model^a^

The quantitative and qualitative data were merged using the lens of the HBM (Fig. 1). Corroborative findings between the two data sets were discovered in the construct of perceived severity and cues to action. For the perceived severity question, the majority of the participants (92–67 %) could identify people who are at increased risk for FBI and were able to identify most of the foods that may increase the risk of FBI for pregnant women, infants, and children with the exception of cold potato salad (19 %) (Table 2). The qualitative theme that corroborated with the quantitative data was “food can make one sick.” From the survey and the focus group discussion, participants preferred participatory classes (cues to action). The discrepant findings uncovered in the constructs of perceived susceptibility and self-efficacy provided information for food safety messaging within this group.

## Discussion

### Perceived Severity

Participants were aware of the severity of FBI as 88 % knew *E. coli* in undercooked hamburger can cause kidney failure in children. One participant aptly described the severity, “I know someone who passed away… *E. coli*… she got ice cubes out of a cooler that they were keeping hamburger patties in and it shut her kidneys down. She was not much older, about eight.” Not only did this statement focus on the severity of foodborne illness but it also indicates the severity for children. Many described stomach cramps and vomiting as effects of FBI on the gastrointestinal tract as verbalized by one participant, “I kept throwing up constantly.” They believed that the gastrointestinal symptoms of a FBI come on quickly after eating and that is how they discern between FBI and the influenza virus. They reported other consequences including lost wages, inconveniences, and the temporary absence of a food preparer.

### Perceived Susceptibility

The theme of food can make one sick emerged as participants discussed perceived susceptibility to getting a FBI. In addition to unsafe handling of foods that can contribute to a FBI, participants identified other features of food that can make one sick. Foods associated with allergies or diet modifications for chronic diseases like diabetes were discussed and illustrated by one participant as “Well with my mom, sugar makes her more sick because she is diabetic.” It is known that Native American adults and youth have 2.3 times and 9 times, respectively, likelihood of a type II diabetes diagnosis compared to non-Hispanic whites [24]. In a qualitative study among Native American women, the primary health concern of diabetes was identified [25]. Others mentioned food intolerances, “like Native Americans, a lot of them are lactose intolerant.” Food allergies were also discussed, “keep a close eye on the child maybe; make sure there was not a reaction of something.” Participants were also more likely to completely avoid what was considered as an unsafe food to prevent them and their family from getting a FBI, “just do not feed it to them.” Clearly, these results demonstrate that food safety was a broad topic to the participants. The bacterial and viral source or etiology of FBI should be identified in food safety messaging to enhance understanding of how the risk is related to proper food handling rather than other constituents in food.

Many individuals were perceived as being susceptible to FBI including children and the elderly. On the food knowledge survey, 89 % knew that preschool children were more likely to get sick from FBI, but one half failed to identify 8 out of 14 foods that increase FBI risk. Some participants felt that they were at a higher risk of obtaining a FBI because the food supply in the USA was not regulated as is should be and because “there is stuff that gets recalled” and “there was a scare on that (cantaloupe) and there was a scare on peanut butter…” However, some participants felt that they were at a low risk of getting a FBI in the USA than in other countries because of food safety regulations. Despite verbalizing that FBI can make one sick, and that preschool children were more likely to get sick from FBI, less than one half correctly identified foods especially likely to be carriers of FBI.

### Perceived Benefits

The focus group script included questions to explore the perceived benefits of food safety; however, the discussions were more consistent with perceived barriers and self-efficacy. One study found that Native Americans strive to be a positive health role model for their children [25]. Participants in this study may have that same vision of benefiting their children as implied by their willingness to participate in a study on food safety and their stated concern for their children to stay healthy.

### Perceived Barriers

Consuming food handled by others was a perceived barrier to avoiding FBI as described by participants, “they may not cook it right” and “who knows if they do what they are supposed to do properly.” Foods prepared at Native American events/feasts were identified as potential sources of FBI. Participants lacked confidence in the food supply from reservation grocery stores, food pantries, and commodity foods. In addition, questionable food safety at sit down and fast food restaurants were described. This concern is supported by a recent report that 68 % of the outbreaks in the USA over 10 years were from restaurants and delis as the single place of food preparation [1].

Other barriers to safe food preparation included lack of time and childcare duties. When studying barriers to behavior change among Native American participants, researchers found lack of time, money, and childcare as barriers [25]. Many participants believed that they need better equipment similar to what they view on television cooking shows to adequately prevent their family from getting sick from FBIs. One participant responded, “when I watch the cooking channels, the stuff for everything they use to cook, at home, I only have the basic stuff” and another, “if I had Rachel Ray’s kitchen.” Stainless steel countertops for ease of cleaning and meat thermometers to determine proper cook temperatures were resources they cited as lacking.

Most participants desired a larger refrigerator for proper food storage. This could be due to the fact that over half of the participants in this study live on reservations that are identified as food desserts according to the US Department of Agriculture [26]. A food dessert is defined by USDA as “urban neighborhoods and rural towns without ready access to fresh, healthy, and affordable food.” Additionally, the rates of tribal households without regular access to a vehicle to travel to a supermarket are higher than found in the US general population. If the participants procure large quantities of perishable foods at one shopping trip, a “larger refrigerator” may be desirable. A significant difference between those living on or off the reservation were found in two questions, “which foods need to be refrigerated to prevent food poisoning” and “how should fresh fruits and vegetables be washed to keep you from getting food poisoning.” It is unknown if responses to the first question is related to limited refrigeration reported by those living on the reservation. Focus group discussions of those living on reservations included concerns about the sanitary condition of reservation grocery stores which may have led to increased food safety knowledge for washing fresh fruits and vegetables. More exploration of these findings is needed in future research.

### Self-Efficacy

Overall, participants were confident in their ability to prepare foods safely for their families which contrasted with the food safety knowledge mean score of 62.2 % (75 % and above was considered an acceptable score). Many believed since no one has “gotten sick from my cooking,” that they are handling food safely. This belief is similar among other main food preparers for young children who have low food safety knowledge [3, 8] and among college participants [27]. Another study reported that professionals in the Supplemental Food Program for Women, Infants, and Children (WIC) strongly believe their clients lack food safety knowledge and need education [28]. Although knowledge is not a part of self-efficacy, knowing the proper food safety practice is a prerequisite to being able to carry out the practice. One example would be using a thermometer for measuring doneness of food. If a person did not know how to use a thermometer, they would not feel confident to carry out this recommended practice.

Improper food safety practices within all four FightBac!^™^ concepts that were discussed in the focus groups aligned with responses on the food safety knowledge survey. Within the clean concept, participants identified hand washing as an overall healthy practice but only 51 % chose the correct hand washing procedure on the food knowledge survey. Sanitizing of baby bottles was discussed only once. It is unknown if this is a common practice among Native Americans. Washing raw poultry and hamburger prior to cooking to remove germs was reported. This practice is not recommended as washing raw meat can cause the juices to splatter onto other surfaces and increases the risk of cross contamination [29]. Mixed descriptions on how bleach is used indicate that education is needed for proper use and to decrease the risk of getting sick from chemical residue as observed from these comments, “I have a cleaning solution, half water, half bleach”; “I use bleach in my dish water soap”; and “just a couple of drops (of bleach).”

Prevalent among improper practices reported within the cook concept was the belief that checking the color of meat to determine doneness is safe as illustrated by the comment of one participant, “they say you need a meat thermometer, but I mean just by looking at it, you can tell if it is red or pink.” In other cases, the meat is overcooked for food safety as described by another participant, “burn them (hamburgers) to get them extra brown.” These practices corroborate with the food safety knowledge survey questions, where less than 40 % of the participants identified that using a thermometer is the best way to tell when chicken or hamburger is cooked long enough to prevent food poisoning.

Within the chill concept, unsafe thawing of frozen meat was described. It is unknown if this is related to the reported need for more refrigeration described earlier. The separate concept, commonly associated with cross contamination, was the least mentioned in the focus groups. Keeping fresh produce separated from raw meats especially on cutting boards was a practice frequently mentioned in the focus groups. Although the concept also includes keeping shell eggs separate from fresh produce, this was not mentioned as a precaution that participants take. More investigation needs to occur with the separate concept among this population.

Findings from other studies parallel the food safety practices and knowledge of Native Americans with young children. Low thermometer use, unsafe thawing of frozen meat, and leaving perishable food at room temperature for longer than 2 h among clients in the Supplemental Food Program for Women, Infants, and Children have been found [6]. Another survey of Australian families with children found poor hand washing, cross contamination of raw meat with cooked meat on cooking boards, and improper thawing of foods [4]. Poor hand washing and thawing methods and lack of food thermometer use among Latino women were observed by other researches [9].

### Cues to Action

Participants previously received health and food safety information from clinics, physicians, classes, health fairs, and media sources including television and the Internet. Participants also reported that they receive a majority of their food preparation education from family members, similar to findings in another study which include a small number of Native Americans [30]. Other researchers reported that Native Americans look to family members and elders for guidance and use traditional methods for medicine and illness such as visiting healers, herbalists, and medicine men instead of physicians [21]. However, participants in our study were interested in participatory classes from a food safety expert accompanied with easy to read and simple printed materials. These findings corroborated from both the survey and focus group discussion. These results support the suggestion of one study that education programs should include “opportunities for participants to come together and share experiences, which is in keeping with the oral traditions of the Native American populations” [25]. Using accepted customs of communication will enhance the delivery of food safety messages [31].

The use of the Health Belief Model to explore food safety attitudes and behaviors of the main food preparers in Native American families with young children ≤10 years of age uncovered several discrepancies when compared to their food safety knowledge. Unknown practices (washing meats and improper use of bleach and using bleach to wash produce) and the broad interpretation to food safety were uncovered by qualitative inquiry. Main food preparers’ perceived that threat to contacting a FBI is potentially high. However, the likelihood of action of consistently practicing safe food handling is potentially low due to barriers expressed coupled with low food safety knowledge measured. Interest in food safety education (cues to action) despite being overly confident in their ability to prepare food safely for their families could indicate that they are not as confident as they verbalized, thus making behavior change possible. Different aspects of the model may be further tested with other groups to understand factors predicting food safety behaviors.

### Limitations

Participants were not randomly selected; therefore, the data cannot be generalized to the Native American population in the USA. The administration of the food safety knowledge survey prior to the focus group exploration may have biased the discussions. A strength of the study was that participants were recruited by key individuals within the Native American population, thus promoting participant trust to discuss beliefs and practices to outside researchers. The use of qualitative research methodology enhanced culturally sensitive communication [32] and uncovered unknown and unsafe food handling practices.

## Conclusion

Native American families with young children had personal experiences with previous FBIs. Participants believed that FBIs could be severe and that they and their families are susceptible to getting FBIs, especially when food is handled by others. Main food preparers were very confident in their ability to prepare food safely for their families but had low food safety knowledge scores. In addition to FightBac!^™^ messages, specific topics for inclusion in food safety education conducted in face to face classes include the bacterial and viral etiologies of FBI and addressing unacceptable practices such as the use of bleach in dishwater to sanitize dishware and washing raw meat prior to preparation. Increased food safety education and best practices for safe food handling are needed among Nebraska Native Americans with young children.
